# Whole-Genome Sequencing of Klebsiella pneumoniae BASUSDALSc45PDB48, a Unique Strain Capable of Growing in Pesticide-Containing Medium, Isolated from Soil in Bangladesh

**DOI:** 10.1128/MRA.00704-21

**Published:** 2021-12-09

**Authors:** Md Atikur Rahman, Sadiya Tahsin, Salek Ahmed Sajib, Mohammad Tanbir Habib, Khaled Mahmud Sujon, Khandaker Md Khalid-Bin Ferdaus, K. M. F. Hoque, Zennat Ferdousi, Md Abu Reza

**Affiliations:** a Molecular Biology and Protein Science Laboratory, Department of Genetic Engineering and Biotechnology, University of Rajshahi, Rajshahi, Bangladesh; SIPBS, University of Strathclyde

## Abstract

We report the draft genome sequence of Klebsiella pneumoniae strain BASUSDALSc45PDB48, isolated from pesticide-contaminated soil; this strain showed the ability to grow in a medium with cypermethrin as the only carbon source. The genome assembly comprised 5,249,704 bp, with 128.17 Ns per 100 kbp, an *N*_50_ value of 5,035,968 bp, a GC content of 57.57%, and 5,349 annotated genes.

## ANNOUNCEMENT

Klebsiella pneumoniae is a Gram-negative, encapsulated, nonmotile, rod-shaped bacterium. It can be found in soil, surface waters, human skin, mouth, intestine, etc. (1, 2). About 30% of all K. pneumoniae strains can fix nitrogen (3). They can also degrade pesticides and thus have high potential for practical application in bioremediation (4).

K. pneumoniae strain BASUSDALSc45PDB was isolated from pesticide-contaminated soil in Netrokona (24.87947 N, 90.70843 E), Bangladesh. The soil (1 g) was added to a 250-ml Erlenmeyer flask containing 100 ml minimal salt (MS) medium supplemented with cypermethrin (500 μg/ml), a commonly used pesticide in Bangladesh. The flask was incubated for 7 days on a rotary shaker (120 rpm) at 37°C. The enrichment culture was streaked onto agar-solidified minimal salt medium (HiMedia, India) supplemented with cypermethrin (500 μg/ml) and incubated at 37°C for 36 h. Finally, a single colony was isolated, and genomic DNA was extracted using the TIANamp bacteria DNA kit (Tiangen Biotech Co. Ltd., Beijing, China) according to the manufacturer’s protocol. Whole-genome sequencing (WGS) and 16S rRNA sequencing were conducted by Invent Technologies Ltd. (Dhaka, Bangladesh). To identify the bacterial strain, the 16S rRNA region was amplified using the forward primer 8F (GAGTTTGATCCTGGCTCAG) and reverse primer 806R (GGACTACHVGGGTWTCTAAT). For taxonomic identification, the 16S sequence was queried against the nonredundant (nr)/nucleotide (nt) database using BLASTN (5) (https://blast.ncbi.nlm.nih.gov/Blast.cgi).

For WGS, a genomic library was constructed for 151-bp paired-end sequencing using the Illumina DNA Prep library preparation kit (catalog number 20018705; San Diego, CA, USA) following the manufacturer’s protocol. The prepared library was sequenced on the Illumina MiSeq platform. Genome sequencing yielded 1,410,224 reads overall, exceeding 52× coverage. The reads were quality checked using FastQC v0.11.9 (https://www.bioinformatics.babraham.ac.uk/projects/fastqc). No adapter sequences were found. Quality control of the raw sequencing data was performed using Trimmomatic v0.39 with default parameters except SLIDINGWINDOW:4:2 (http://www.usadellab.org/cms/?page=trimmomatic) (6). *De novo* assembly using SPAdes v3.14.1 (https://cab.spbu.ru/software/spades) (7) initially yielded 565 contigs with an *N*_50_ value of 147,594 bp. For the SPAdes assembly, the coverage threshold was calculated automatically using “--cov-cutoff auto,” and the k-mer sizes were set to “-k 21,33,55,77,” while the “--careful” option was selected to minimize the number of mismatches in the final contigs. The resulting assembly was further mapped to the reference genome K. pneumoniae subsp. *pneumoniae* HS11286 (GenBank accession number NC_016845.1) using the scaffolding software RagTag v1.0.1 (https://github.com/malonge/RagTag), which produced 128.17 Ns per 100 kbp (6,600 Ns in total) and 420 scaffolds, with an *N*_50_ value of 5,035,968 bp (8). PlasmidFinder v2.0 (https://cge.cbs.dtu.dk/services/PlasmidFinder/) identified contig 288 as a single complete plasmid (IncN) (9). Default parameters were used for all software unless otherwise specified. The draft genome was annotated using the NCBI Prokaryotic Genome Annotation Pipeline (PGAP) (10, 11). After scaffolding and gap estimation, the Klebsiella pneumoniae strain BASUSDALSc45PDB48 genome assembly size was 5,256,304 bp, with 5,249,704 bp of ungapped sequences (GC content, 57.57%), containing 5,195 protein coding genes, 70 tRNAs, 3 rRNAs, 16 noncoding RNAs, and a single plasmid.

### Data availability.

The K. pneumoniae strain BASUSDALSc45PDB48 genome sequence has been deposited at GenBank under accession numbers JAEKPA000000000.1 (genome) and JAEKPA010000277.1 (plasmid), BioProject accession number PRJNA685932, BioSample accession number SAMN17101190, and SRA accession number SRR13447672.
